# The Aggregation Inhibitor Peptide QBP1 as a Therapeutic Molecule for the Polyglutamine Neurodegenerative Diseases

**DOI:** 10.4061/2011/265084

**Published:** 2011-06-30

**Authors:** H. Akiko Popiel, James R. Burke, Warren J. Strittmatter, Shinya Oishi, Nobutaka Fujii, Toshihide Takeuchi, Tatsushi Toda, Keiji Wada, Yoshitaka Nagai

**Affiliations:** ^1^Department of Degenerative Neurological Diseases, National Institute of Neuroscience, National Center of Neurology and Psychiatry, 4-1-1 Ogawa-Higashi, Kodaira, Tokyo 187-8502, Japan; ^2^Department of Medicine (Neurology) and Deane Laboratory, Duke University Medical Center, Durham, NC 27710, USA; ^3^Department of Bioorganic Medicinal Chemistry, Kyoto University Graduate School of Pharmaceutical Sciences, Kyoto 606-8501, Japan; ^4^Division of Neurology/Molecular Brain Science, Kobe University Graduate School of Medicine, Kobe 650-0017, Japan; ^5^Core Research for Evolutional Science and Technology (CREST), Japan Science and Technology Agency, Saitama 332-0012, Japan

## Abstract

Misfolding and abnormal aggregation of proteins in the brain are implicated in the pathogenesis of various neurodegenerative diseases including Alzheimer's, Parkinson's, and the polyglutamine (polyQ) diseases. In the polyQ diseases, an abnormally expanded polyQ stretch triggers misfolding and aggregation of the disease-causing proteins, eventually resulting in neurodegeneration. In this paper, we introduce our therapeutic strategy against the polyQ diseases using polyQ binding peptide 1 (QBP1), a peptide that we identified by phage display screening. We showed that QBP1 specifically binds to the expanded polyQ stretch and inhibits its misfolding and aggregation, resulting in suppression of neurodegeneration in cell culture and animal models of the polyQ diseases. We further demonstrated the potential of protein transduction domains (PTDs) for *in vivo* delivery of QBP1. We hope that in the near future, chemical analogues of aggregation inhibitor peptides including QBP1 will be developed against protein misfolding-associated neurodegenerative diseases.

## 1. Introduction

**Figure 1:**
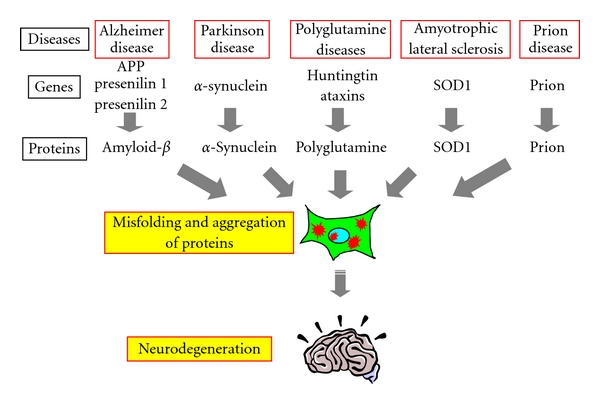
Misfolding and abnormal aggregation of proteins as a common molecular pathogenesis of the protein misfolding diseases. The genetic mutations responsible for the inherited forms of various neurodegenerative diseases render the proteins prone to misfold and aggregate, or lead to the overproduction of aggregation-prone proteins, which accumulate as inclusions inside and outside neurons in the diseased brains, and eventually cause neurodegeneration. These facts indicate that the misfolding and abnormal aggregation of proteins are crucial in the pathogenesis of these diseases, which are known as the “protein misfolding diseases.”

Neurodegenerative diseases are a group of disorders, which are caused by progressive degeneration of neurons in various areas of the brain specific for each disorder, resulting in various neurological and psychiatric symptoms corresponding to each affected brain area. Few effective therapies have been established to date for these diseases, largely due to the fact that the underlying cause of the neurodegeneration long remained unknown. However, accumulating evidence now indicates that many of these neurodegenerative diseases, including Alzheimer's disease (AD), Parkinson's disease (PD), the polyglutamine (polyQ) diseases, amyotrophic lateral sclerosis, and the prion diseases, share a common pathomechanism (Figure 1). Pathological and biochemical studies have revealed that various protein inclusions accumulate inside and outside of neurons in the diseased brains, such as senile plaques composed of amyloid-*β* and neurofibrillary tangles composed of tau in AD, and Lewy bodies composed of *α*-synuclein in PD. Although the significance of these protein inclusions on disease pathology long remained controversial, recent molecular genetics studies revealed that the mutations responsible for the inherited forms of these diseases render the proteins to be prone to misfold and aggregate, or lead to the overproduction of aggregation-prone proteins. Furthermore, not only such genetic mutations, but also multiple environmental factors are thought to trigger the misfolding of otherwise normal proteins, and indeed the sporadic cases of these diseases also exhibit similar protein inclusions in the brain. It is noteworthy that the aggregates composed of different proteins accumulated in the different diseases all have a similar structure, namely, that they are *β*-sheet-rich amyloid. In addition, genetic animal models expressing these aggregation-prone mutant proteins have been found to develop similar protein inclusions as well as neurodegeneration. These facts, taken together, have strongly suggested that the misfolding and abnormal aggregation of proteins are crucial in the pathogenesis of these neurodegenerative diseases, which are hence collectively called the “protein misfolding diseases” [1–3] (Figure 1). 

Our group has been working towards establishing therapies for these protein misfolding diseases, with a particular focus on the polyQ diseases because of the following reasons. Firstly, they are determined almost solely by a monogenic mutation and are minorly influenced by environmental factors unlike the other diseases. Furthermore, there is a tight correlation between the severity of the genetic mutation and the disease phenotypes. These special characteristics highlight the polyQ diseases as the most suitable model for the protein misfolding diseases.

In this review, we will introduce our research towards establishing a therapy for the polyQ diseases by targeting the protein misfolding and aggregation, using polyglutamine binding peptide 1 (QBP1), a small biologically active peptide that we identified from combinatorial screening.

## 2. The Polyglutamine Diseases

Molecular genetics studies on inherited neurodegenerative diseases in the last few decades have revealed a common genetic mutation shared by a group of diseases, namely, an expansion (>40) of the CAG repeat encoding a polyQ stretch in each unrelated disease-causing gene, and hence these diseases are called the polyQ diseases [4, 5]. Currently nine diseases have been found to belong to this group, including Huntington's disease, spinocerebellar ataxia (SCA) type 1, 2, 3, 6, 7, and 17, dentatorubral pallidoluysian atrophy, and spinobulbar muscular atrophy (SBMA) [6–17].

The polyQ diseases share many common characteristics, although the responsible proteins share no particular functional or sequence similarities except for the polyQ stretch. Most of the diseases are inherited through an autosomal dominant manner except for SBMA. The threshold of the polyQ repeat size for disease manifestation is approximately 35–40, except for SCA6, and the length of the polyQ repeat is tightly correlated with the age of onset and severity of the disease. These facts taken together strongly indicate that the expanded polyQ stretch itself causes these diseases via a gain of toxic function mechanism, which is unrelated with the normal function of the host protein. Indeed, expression of an expanded polyQ stretch alone or even an expanded polyQ stretch introduced into an unrelated protein has been shown to cause neurodegeneration in various experimental animal models [18–21].

**Figure 2:**
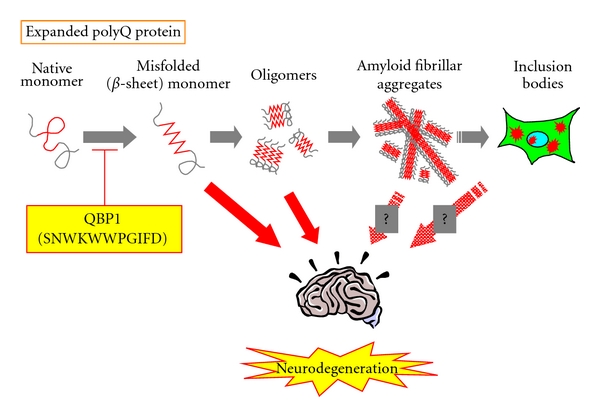
Molecular pathogenesis of the polyQ diseases and the therapeutic target of QBP1. Proteins with an expanded polyQ stretch are prone to misfold into a *β*-sheet dominant structure, leading to their assembly into oligomers and amyloid fibrillar aggregates, followed by their accumulation as inclusion bodies within neurons, eventually resulting in neurodegeneration. The peptide QBP1 inhibits the initial misfolding into a *β*-sheet dominant structure of the protein by binding to the expanded polyQ stretch, resulting in suppression of polyQ protein aggregation and polyQ-induced neurodegeneration. Question marks indicate structures for which cytotoxicity remains controversial.

As a common molecular pathogenesis of the polyQ diseases, it has been proposed that proteins with an expanded polyQ stretch become misfolded and form oligomers and amyloid fibrillar aggregates, and subsequently accumulate as inclusion bodies within neurons, eventually resulting in neurodegeneration (Figure 2) [22–26]. Various cellular proteins have been shown to associate with the polyQ aggregates/inclusion bodies, including transcription factors [27, 28], molecular chaperones [29, 30], cytoskeletal proteins [31], and proteasomal subunits [29], and such abnormal associations are thought to play a role in the disease pathogenesis, through dysfunction of the cellular processes involving these proteins. Accordingly, there have been therapeutic approaches targeting each specific cellular process that is compromised in the disease pathogenesis [23, 32]. However, these attempts result in only limited therapeutic effects, since numerous cellular processes are affected by expression of the expanded polyQ protein [33–36]. In contrast to these downstream events, misfolding and aggregation of the expanded polyQ proteins are the most initial events of the pathogenic cascade, and therefore ideal targets since their intervention is expected to lead to the suppression of a broad range of downstream pathogenic events [22, 24, 37, 38]. We therefore aimed towards establishing a therapy targeting misfolding and aggregation of the expanded polyQ protein.

## 3. Identification of the Aggregation Inhibitor Peptide QBP1

**Figure 3:**
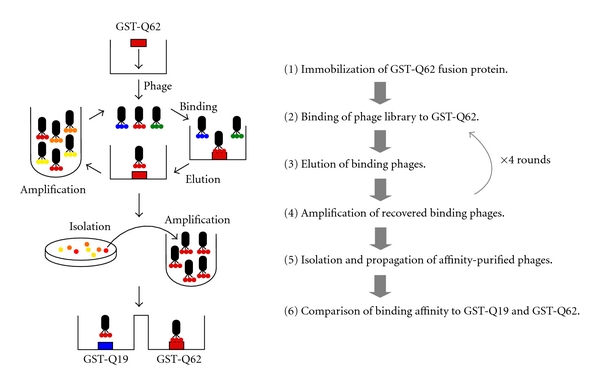
Phage display screening strategy for the identification of peptides that selectively bind to the expanded polyQ stretch. Phage libraries expressing random 11-amino acid sequences were first screened for their binding to GST-Q62 via 4 rounds of binding, elution, and amplification. Phage clones isolated from the first screening (350 clones) were further screened for their selective binding to pathologic length GST-Q62 compared to normal-length GST-Q19.

We hypothesized that molecules capable of binding specifically to the expanded polyQ stretch would interfere with its misfolding and aggregation. Identification of the monoclonal antibody 1C2 that selectively binds to the expanded polyQ stretch, probably by recognizing its unique structure [39], prompted us to search for amino acid sequences (domains) or peptides possessing similar properties, which would be more suitable as a drug due to their smaller size and more efficient *in vivo* delivery. We decided to employ phage display screening to identify peptides that bind selectively to the expanded polyQ stretch (Figure 3) [40]. Eleven-amino acid combinatorial peptide libraries expressed on the surface of M13 phage were first screened for their binding to a polyQ62 stretch fused to glutathione *S*-transferase (GST-Q62) by enzyme immunosorbent assay. Phage clones isolated from this first screening were further screened for their selective binding to pathologic length GST-Q62 compared to normal-length GST-Q19. We finally identified six phage clones with greater binding to GST-Q62, and named the encoded peptide sequences polyglutamine binding peptide 1-6 (QBP1-6) (Table 1). Interestingly, most of the peptides were rich in Trp residues, implying that hydrophobic interactions play a role in their binding to the expanded polyQ stretch. We chose QBP1 (Ser-Asn-Trp-Lys-Trp-Trp-Pro-Gly-Ile-Phe-Asp), which showed the greatest differential binding affinity to pathologic length polyQ compared with normal length polyQ for further analysis. 

We first tested our hypothesis that QBP1, a peptide that selectively binds to the expanded polyQ stretch would interfere with polyQ aggregation *in vitro* [40]. We designed thioredoxin-polyQ (thio-polyQ) fusion proteins, and found that thio-polyQ with an expanded polyQ stretch (>40) forms aggregates *in vitro *in a time-, concentration-, and polyQ length-dependent manner, which faithfully mimic the *in vivo* characteristics of disease-causing polyQ proteins. We coincubated QBP1 with thio-Q62, and found that QBP1 dramatically inhibits thio-Q62 aggregation in a concentration-dependent manner, showing an almost complete inhibition at a stoichiometry of 3 : 1 (thio-Q62 : QBP1). A scrambled sequence of QBP1 (SCR; Trp-Pro-Ile-Trp-Ser-Lys-Gly-Asn-Asp-Trp-Phe) had no effect on thio-Q62 aggregation. Furthermore, addition of QBP1 after thio-Q62 aggregation has started resulted in inhibition of further aggregate formation, but it could not solubilize the aggregates already formed, suggesting that QBP1 inhibits the earlier stages in the aggregation process of the expanded polyQ protein [41].

## 4. Mechanism of Action of QBP1

To elucidate the molecular mechanisms by which QBP1 prevents aggregation of the expanded polyQ protein, we have characterized in detail the binding of QBP1 to the expanded polyQ stretch, and analyzed the effect of QBP1 on the conformation of the expanded polyQ protein. To characterize the binding specificities and affinities of QBP1 to the polyQ stretch, we employed the surface plasmon resonance (SPR) technique, which is a highly sensitive method for quantitatively measuring biomolecular interactions [42]. We found that QBP1 binds selectively to the thio-Q62 protein, with an equilibrium dissociation constant (*K*_*d*_) of 5.7 *μ*M, while it shows significant binding to neither thio-Q0 nor thio-Q19. These results clearly indicate the striking property of QBP1 to specifically recognize and bind to the expanded polyQ stretch, but not the normal length polyQ stretch. We also investigated the relationship between the polyQ binding affinities of QBP1 and its variants and their inhibitory effects on polyQ aggregation. We found a tight correlation between the binding affinities to the expanded polyQ stretch and inhibitory activities on polyQ aggregation of these peptides. Among these, (QBP1)_2_, a tandem repeat of QBP1 exhibited the greatest binding affinity to thio-Q62 with a *K*_*d*_ value of 0.6 *μ*M. We therefore conclude that binding affinity to the polyQ stretch is a critical determinant of the aggregation inhibitory activity. 

Next, we analyzed the effect of QBP1 on the conformation of the expanded thio-polyQ protein [43]. Circular dichroism (CD) analyses revealed that QBP1 inhibits the conformational transition of the thio-Q62 protein to a *β*-sheet dominant structure. We further demonstrated for the first time that this *β*-sheet conformational transition of the expanded polyQ protein, which occurs at the level of the monomer before aggregation, causes cytotoxicity. Taken together, we conclude that QBP1 specifically binds to the expanded polyQ protein monomer and inhibits the toxic *β*-sheet conformational transition, and as a result, also inhibits the downstream aggregation and inclusion body formation (Figure 2).

## 5. The Therapeutic Effects of QBP1 Expression in Cell Culture Models of the PolyQ Diseases

We also determined whether QBP1 could exert therapeutic effects in cell culture models of the polyQ diseases [40]. Expanded polyQ proteins expressed in cultured cells have been shown to form inclusion bodies and cause cytotoxicity in a time- and polyQ length-dependent manner [44]. We first coexpressed QBP1 fused to cyan fluorescent protein (QBP1-CFP), with various-length polyQ proteins fused to yellow fluorescent protein (polyQ-YFP) in COS-7 cells, and examined the effect of QBP1 on polyQ inclusion body formation and cytotoxicity. We found a prominent colocalization of QBP1-CFP with polyQ-YFP inclusions, indicating that QBP1 is capable of recognizing the polyQ stretches in cells. Notably, coexpression of QBP1-CFP significantly suppressed polyQ-YFP inclusion body formation, as well as cytotoxicity, and the inhibitory effects were stronger for shorter-length polyQ stretches (Q45 > Q57 > Q81). Furthermore, (QBP1)_2_-CFP, which has a much higher affinity to the expanded polyQ stretch, exerted an even stronger inhibitory effect on polyQ inclusion body formation, consistent with our *in vitro* aggregation assay results [42]. 

The expanded polyQ protein is recently believed to form soluble oligomers before microscopically visible insoluble aggregates and inclusion bodies in cells, and these oligomers rather than aggregates or inclusion bodies have been suggested to cause cytotoxicity [24] (Figure 2). We therefore analyzed the effect of QBP1 on polyQ oligomer formation, by using fluorescence correlation spectroscopy (FCS), which is a highly sensitive technique for investigating the dynamics of fluorescent molecules at single molecule sensitivity [45]. We found that the time-dependent decrease in mobility and increase in size of the expanded polyQ-green fluorescent protein (polyQ-GFP) expressed in COS-7 cells, which indicates the formation of slowly moving oligomers, was significantly suppressed by the coexpression of (QBP1)_2_-CFP [46]. Fluorescence resonance energy transfer (FRET) analyses also confirmed that (QBP1)_2_ inhibits expanded polyQ oligomer formation in cultured cells [47]. These results are consistent with our *in vitro* observation that QBP1 inhibits the conformational transition of the polyQ protein monomer, which occurs before oligomer and aggregate formation.

## 6. Therapeutic Effects of QBP1 Expression in Animal Models of the PolyQ Diseases

From a therapeutic viewpoint, it is indispensable to demonstrate the therapeutic effect of QBP1 in *in vivo* disease models. We employed *Drosophila* models to elucidate the therapeutic effects of QBP1 expression on polyQ-induced neurodegeneration, since *Drosophila* models of the polyQ diseases are well established, easy to handle, and suitable for genetic analyses [48]. Transgenic flies expressing an expanded polyQ protein under an eye-specific promoter demonstrate accumulation of polyQ inclusion bodies and degeneration of the eyes. We crossed polyQ expressing flies and (QBP1)_2_-CFP expressing flies, and found that coexpression of (QBP1)_2_-CFP significantly suppresses eye degeneration, as well as inclusion body formation. We next examined the effect of (QBP1)_2_-CFP coexpression on flies expressing the expanded polyQ protein under a panneuronal promoter, which causes premature death due to neurodegeneration. Notably, the median life span of polyQ expressing flies was dramatically improved from 5.5 days to 52 days by coexpression of (QBP1)_2_-CFP, indicating that QBP1 successfully rescues premature death of the polyQ flies. These results clearly demonstrate the effectiveness of QBP1 on polyQ-induced neurodegeneration *in vivo*.

## 7. Therapeutic Effects of Protein Transduction Domain-Mediated Delivery of QBP1

To establish a therapy using QBP1, QBP1 needs to be delivered into affected neurons in the brain, rather than expressed by the crossing of genetically engineered animals. However, as QBP1 is an 11-amino acid peptide, it is too large to cross the cell membrane efficiently and enter cells on its own. To enable the efficient intracellular delivery of QBP1, we utilized protein transduction domains (PTDs), which are peptide sequences capable of penetrating the cell membrane and entering cells. These include the human immunodeficiency virus-1 TAT, *Drosophila* Antennapedia (Antp), herpes simplex virus-1 VP22, and the polyarginines (Table 2). PTDs have indeed been shown to efficiently deliver various biologically active molecules such as peptides, proteins, and nucleic acids into cells [49, 50]. 

We synthesized QBP1 peptides fused to the TAT or Antp PTD, and confirmed that both of them are efficiently transduced into cells upon addition to the medium of cultured cells, and inhibit inclusion body formation and cytotoxicity of the expanded polyQ protein [51]. To determine whether PTD-QBP1 administration is able to exert therapeutic effects on an *in vivo* model of the polyQ diseases, we first administered Antp-QBP1 to a *Drosophila* polyQ disease model, by adding the peptide into the culture food. Oral administration of Antp-QBP1 remarkably delayed premature death of the polyQ expressing flies compared with the control peptide Antp-SCR. In addition, flies administered with Antp-QBP1 had significantly fewer inclusion bodies compared to the control flies. These results indicated the potential of PTD-mediated delivery of QBP1 as a useful strategy to establish a molecular therapy using QBP1. 

We next analyzed the therapeutic effect of Antp-QBP1 administration on a mouse model of the polyQ diseases [52]. Intraperitoneal injection of Antp-QBP1 resulted in a slight improvement of the weight loss in these mice, but did not improve the other phenotypes such as motor dysfunction and premature death. Furthermore, we could not detect a significant suppression of polyQ inclusion body formation by Antp-QBP1 administration in these mice. Although we confirmed the limited delivery of Antp-QBP1 into the mouse brain via intracerebroventricular and intrastriatal injection, we failed to detect a significant amount of Antp-QBP1 delivered in the brain via intraperitoneal injection. These results imply that Antp-QBP1 is unable to efficiently cross the blood-brain barrier (BBB) in mice, which is tighter than in flies.

## 8. Towards Designing Chemical Analogues of QBP1

Towards developing QBP1 as a therapeutic molecule for the polyQ diseases, we are taking another approach, which is designing low molecular weight chemical analogues of QBP1 with efficient BBB permeability. To design low molecular weight analogues of QBP1, we first determined the essential amino acids required for its activity and pharmacophores of QBP1. 

We first synthesized various truncation mutants of QBP1, and tested their activities on polyQ aggregation. We found that truncation of Ser1 and Asn2, or truncation of Asp11 does not affect the inhibitory activity on polyQ aggregation whereas truncation of the N-terminal 4 amino acids (Ser1, Asn2, Trp3, and Lys4), or the C-terminal 2 amino acids (Phe10 and Asp11) results in dramatic loss of activity. These results imply that the aromatic amino acids (Trp3 and Phe10) are required for the activity of QBP1, and we therefore concluded that the central 8 amino acids (Trp-Lys-Trp-Trp-Pro-Gly-Ile-Phe) comprise the minimal active sequence of QBP1 [41]. Since other QBPs that we identified from the combinatorial screening also share Trp/Phe-rich sequences (Table 1), we next investigated the role of the Trp-Lys-Trp-Trp motif of QBP1 for its activity. Although the Trp-Lys-Trp-Trp motif alone is insufficient for inhibiting polyQ aggregation, a tandem repeat of Trp-Lys-Trp-Trp connected by an amino acid spacer was found to be as potent as the original QBP1, suggesting that the Trp-Lys-Trp-Trp motif plays an important role in recognizing the polyQ stretch. 

We subsequently performed more comprehensive analyses on all amino acids within the QBP1 sequence by Ala scanning and D-amino acid scanning [53]. Substitutions of Ser1, Asn2, Lys4, Pro7, Gly8, or Asp11 to Ala did not show any significant effects on the activity of QBP1. On the other hand, Ala substitutions of Trp3, Trp5, Trp6, Ile9, or Phe10 led to a dramatic decrease in their activity on polyQ aggregation, indicating that the functional groups of these hydrophobic amino acids are essential for their inhibitory activity. Hence, the hydrophobic property of QBP1 may be important for its interaction with the expanded polyQ stretch. In addition, D-amino acid substitutions revealed that the internal amino acids (Trp3-Ile9) of QBP1 are sensitive to chirality inversion, which probably disrupts the active conformation of QBP1. 

Another study using a series of peptide analogues of QBP1 elucidated the role of the Trp residues in the activity of QBP1 [54]. Although N-methylation at the main chain of Trp5 and Trp6, which would lose their potential as main chain hydrogen bond donors, resulted in a substantial loss of activity of QBP1, methylation of the indole nitrogen of these residues did not affect its activity, suggesting that the hydrogen bonding potential of the indole side chains are not necessary for the activity of QBP1. 

In order to design chemical analogues of QBP1, it is also indispensable to obtain structural information on the mode of binding of QBP1 to the polyQ stretch. However, due to the high insolubility of the expanded polyQ protein, it has been a challenge to experimentally elucidate the structure of the polyQ stretch at atomic resolution in aqueous solution. Although a molecular dynamics study suggested hydrogen bonding between the amide groups of Ser1 to Gly8 of QBP1 and the main chain carbonyl groups of the polyQ stretch, and the role of the steric hindrance produced by Pro7 to prevent polyQ aggregation [55], there are some inconsistencies with the experimental results described above. Thus, further efforts to elucidate the detailed structure of the QBP1-polyQ complex would provide valuable information for designing chemical QBP1 analogues as a therapeutic molecule for the polyQ diseases.

## 9. Other Applications of QBP1

Since QBP1 is the only molecule which can distinguish between the expanded and normal length polyQ stretch, it is also useful for specific recognition of the expanded polyQ stretch. Indeed, we have confirmed the colocalization of QBP1 with polyQ inclusions [40], and recently, Raspe et al. also utilized QBP1-CFP to label expanded polyQ peptides within inclusion bodies in cultured cells [56]. These studies raise the possibility that QBP1 could also be developed as an *in vivo* imaging probe for detection of polyQ depositions in the brain. 

Bauer et al. also employed QBP1 to recognize expanded polyQ proteins for their specific degradation by chaperone-mediated autophagy (CMA), in which Hsc70 recognizes and delivers substrate proteins to the lysosome for their degradation [57]. Coexpression of a modified QBP1, which was fused with Hsc70-binding motifs, with expanded polyQ proteins accelerated polyQ protein degradation, resulting in suppression of cytotoxicity in cultured cells. They further demonstrated that viral vector-mediated gene therapy of the modified QBP1 decreased polyQ protein aggregation and ameliorated phenotypes such as motor dysfunction and premature death in polyQ disease mice while viral expression of the original QBP1 alone also exhibited a modest therapeutic effect. These results clearly indicate the usefulness of QBP1 as a tool for specific recognition of the expanded polyQ protein.

## 10. Other Peptides/Proteins that Bind to PolyQ and Inhibit Aggregation

Discovery of QBP1 has facilitated research towards applying various polyQ-binding molecules such as peptides and proteins to prevent misfolding and aggregation of the expanded polyQ protein like as QBP1. Kazantsev et al. designed a bivalent peptide comprised of two normal-length polyQ stretches connected by a spacer, which is expected to bind to the expanded polyQ stretch, and showed that expression of this peptide suppresses polyQ inclusion body formation and cytotoxicity in cell culture and *Drosophila *polyQ disease models [58]. We also designed a normal-length polyQ stretch with a Pro insertion, which disrupts the ordered structure of the polyQ stretch, and showed that this peptide successfully delays polyQ aggregation* in vitro *[59]. However, since these rationally designed peptides contain short polyQ stretches that can be recruited to expanded polyQ aggregates, they have the risk of accelerating polyQ aggregation and enhancing toxicity under certain conditions. Furthermore, the therapeutic effects of these peptides were much weaker compared to QBP1, which is the optimal peptide sequence identified by a combinatorial screening approach for its specific binding affinity to the expanded polyQ stretch, and is the only molecule that has been shown to inhibit the toxic *β*-sheet conformational transition of the expanded polyQ protein [43]. 

Several intracellular antibodies, known as intrabodies, which bind to the expanded polyQ protein and inhibit its aggregation have also been identified to date. In 2001, Lecerf et al. identified the intrabody C4 that binds to the N-terminus of huntingtin (htt), the disease-causing protein of Huntington's disease (HD), by phage display library screening [60]. Subsequently, they and other groups further showed that expression of C4 as well as other intrabodies, namely, MW7, V_L_12.3, Happ1, and EM48, all of which bind to the polyQ adjacent regions in htt, leads to suppression of htt aggregation and neurodegeneration in cell culture, *Drosophila*, and mouse models of HD [60–67]. The use of intrabodies is an attractive therapeutic approach with regard to their high binding affinity to the disease-causing proteins. However, since the intrabodies identified so far recognize a region in htt other than the polyQ stretch itself, they cannot be applied for the other polyQ diseases, and may cause unfavorable side effects by binding to the wild type htt with a normal polyQ stretch.

## 11. Perspectives

In this review, we introduced our therapeutic strategy against the polyQ neurodegenerative diseases using QBP1, a peptide sequence that specifically recognizes the expanded polyQ stretch, which we identified from phage display screening. Although we have provided convincing evidence on the potential of QBP1 as a therapy for the polyQ diseases, by demonstrating its ability to inhibit misfolding and aggregation, resulting in suppression of polyQ-induced neurodegeneration *in vivo*, the major problem we are currently facing is its delivery into the brain. Although viral vector-mediated gene therapy may have potential for the delivery of QBP1 into the brain, the difficulty in controlling gene expression, toxicity, and limited delivery within the brain discourage this approach. The success of PTD-mediated delivery of QBP1 and its therapeutic effects in a* Drosophila* model of the polyQ diseases have shed light on the potential of PTDs for *in vivo* delivery of QBP1. Recently, an unconventional secretion signal overlapped with the Antp sequence was identified, which enables secretion from cells in addition to entry into cells via Antp [68], suggesting the potential of identifying or designing novel PTDs with high BBB permeability. Since most therapeutic molecules currently in clinical use are chemical compounds, we believe the most promising approach is to design low molecular weight chemical QBP1 analogues with efficient BBB permeability. Further clarification of the mode of binding of QBP1 to the expanded polyQ stretch and detailed structural analyses of the QBP1-polyQ complex will facilitate the designing of chemical analogues of QBP1 as a potential therapeutic molecule for the polyQ diseases. 

Although our work has been focused on the polyQ diseases, our approach could also be applied for a broad range of other neurodegenerative diseases including Alzheimer's disease and Parkinson's disease, which are caused by a common mechanism based on protein misfolding and aggregation. Indeed, various peptides/proteins that inhibit protein aggregation have been reported to exert therapeutic effects in cell culture and animal models of these diseases [69, 70]. We hope that in the near future, aggregation inhibitor peptide-based drugs against protein misfolding neurodegenerative diseases will be developed and bring a cure to patients suffering from these currently intractable neurodegenerative diseases.

## Figures and Tables

**Table 1:** Polyglutamine binding peptides isolated from phage display screening.

Name	Q62/Q19 binding ratio	Sequence (X_5_-fixed-X_5_)
QBP1	1.66	Ser-Asn-Trp-Lys-Trp-Trp-Pro-Gly-Ile-Phe-Asp
QBP2	1.31	His-Trp-Trp-Arg-Ser-Trp-Tyr-Ser-Asp-Ser-Val
QBP3	1.30	His-Glu-Trp-His-Trp-Trp-His-Gln-Glu-Ala-Ala
QBP4	1.27	Trp-Gly-Leu-Glu-His-Phe-Ala-Gly-Asn-Lys-Arg
QBP5	1.25	Trp-Trp-Arg-Trp-Asn-Trp-Ala-Thr-Pro-Val-Asp
QBP6	1.23	Trp-His-Asn-Tyr-Phe-His-Trp-Trp-Gln-Asp-Thr
SCR		Trp-Pro-Ile-Trp-Ser-Lys-Gly-Asn-Asp-Trp-Phe

**Table 2:** Examples of protein transduction domains.

Name	Origin/design	Sequence
TAT	HIV-1 transactivator	Tyr-Gly-Arg-Lys-Lys-Arg-Arg-Gln-Arg-Arg-Arg
Antp	*Drosophila* Antennapedia	Arg-Gln-Ile-Lys-Ile-Trp-Phe-Gln-Asn-Arg-Arg-Met- Lys-Trp-Lys-Lys
VP22	HSV-1 structural protein	Asp-Ala-Ala-Thr-Ala-Thr-Arg-Gly-Arg-Ser-Ala-Ala-Ser-Arg-Pro-Thr-Glu-Arg-Pro-Arg-Ala-Pro-Ala-Arg- Ser-Ala-Ser-Arg-Pro-Arg-Arg-Pro-Val-Asp
Polyarginine	Synthetic	(Arg)_*n*_
Transportan	Neuropeptide galanin + wasp peptide mastoparan	Gly-Trp-Thr-Leu-Asn-Ser-Ala-Gly-Tyr-Leu-Leu-Gly-Lys-Ile-Asn-Leu-Lys-Ala-Leu-Ala-Ala-Leu-Ala-Lys- Lys-Ile-Leu
